# Role of *hsp20* in the Production of Spores and Insecticidal Crystal Proteins in *Bacillus thuringiensis*

**DOI:** 10.3389/fmicb.2019.02059

**Published:** 2019-09-04

**Authors:** Junyan Xie, Jinli Peng, Zixian Yi, Xiaoli Zhao, Shuiming Li, Tong Zhang, Meifang Quan, Shuqing Yang, Jiaoyang Lu, Pengji Zhou, Liqiu Xia, Xuezhi Ding

**Affiliations:** ^1^State Key Laboratory of Developmental Biology of Freshwater Fish, Hunan Provincial Key Laboratory of Microbial Molecular Biology, College of Life Science, Hunan Normal University, Changsha, China; ^2^Shenzhen Key Laboratory of Microbial Genetic Engineering, College of Life Sciences and Oceanography, Shenzhen University, Shenzhen, China

**Keywords:** *hsp20*, *Bacillus thuringiensis*, sporulation, insecticidal crystal proteins, iTRAQ

## Abstract

The small heat shock protein plays an important role in response to stresses. We wanted to investigate how Hsp20 affects sporulation and production of insecticidal crystal proteins (ICPs) in *Bacillus thuringiensis* (Bt) at the stationary growth phase when cells are starved. The *hsp20* gene was knocked out in Bt4.0718 (wide type), which is a *B. thuringiensis* strain screened in our laboratory, using endonuclease I-SceI mediated unmarked gene replacement method. Deletion of Hsp20 resulted in a decrease in both sporulation and ICPs production. Bt4-Δ*hsp20* cells and its ICP did not have a significant difference in shape and size but entered the decline phase 2 h earlier than the Bt4.0718. In order to find the mechanism that underlies these phenotypes, we completed a proteomic study of differentially expressed proteins (DEPs). In Bt4-Δ*hsp20* cells, 11 DEPs were upregulated and 184 DEPs downregulated. These affected DEPs are involved in multiple metabolic pathways: (1) six DEPs (two upregulated and four downregulated) are directly related to the sporulation and ICPs synthesis; (2) supply of amino acids including amino acid synthesis and protein recycling; (3) the energy supplementation (the tricarboxylic acid cycle and glycolysis); (4) purine metabolism and mRNA stability. These results suggest that *hsp20* may be critical in maintaining the homeostasis of *B. thuringiensis* during the production of spores and ICPs, and could provide new sight into the sporulation and ICPs formation in *B. thuringiensis*.

## Introduction

Heat shock proteins (HSPs) are a family of proteins that are produced in response to stressful conditions, providing a chaperone function by stabilizing proteins to ensure correct folding, or by helping to refold proteins that are damaged by cell stress (Maio, 1999). HSPs exist in bacteria and in humans and are named according to their molecular weight, such as Hsp60, Hsp70, and Hsp90. These HSPs are the most extensively studied HSPs in humans and plants. The expression of HSPs is transcriptionally regulated and triggered by different environmental stresses, such as infections, inflammation, toxins, starvation, hypoxia, and water deprivation. The principal HSPs include five conserved classes, which are HSP33, HSP60, HSP70, HSP90, and HSP100, and small HSPs (sHSPs) (Schlesinger, 1990).

Small Heat shock proteins are ubiquitous ATP-independent molecular chaperones that prevent protein aggregation upon stress-induced unfolding in nearly all organisms (Bepperling et al., 2012). Hsp20 responds to heat shock or other environmental stresses (Burdon, 1988). Numerous studies have confirmed that Hsp20 plays an important role in the response to chemical or physical conditions. For example, the transcriptional level of *hsp20* is strongly induced to resist severe heat and osmotic shocks (Ventura et al., 2007; Khaskheli et al., 2015). In *Escherichia coli* cells, overexpression of Hsp20 of *Deinococcus radiodurans*, and GrxC, IscA, and Fdx proteins are involved in the control of cellular redox status, through enhancing tolerance to hydrogen peroxide (H_2_O_2_) stress (Singh et al., 2014). Under the stress of water deprivation, Hsp20 is essential for the desiccation resistance of *Azotobacter vinelandii* (Cocotl-Yañez et al., 2014). Proteomics analysis of the probiotic bacterium, *Lactobacillus casei*, indicated that Hsp20 may play a crucial role in the adaptation to the physiological processes of lactic acid accumulation (Wu et al., 2009).

Bt, a Gram-positive bacterium and one of the safest microbial pesticides, has been used worldwide in pest control for several decades (Jouzani et al., 2017). It forms characteristic parasporal insecticidal crystal proteins during sporulation (Roh et al., 2007; Van, 2009; Palma et al., 2014; Singh et al., 2018). Moreover, this bacterium can promote plant growth (Armada et al., 2018), bioremediate different heavy metals, biosynthesize metal nanoparticles (Jouzani et al., 2017), produce melanin (Cao et al., 2018), and even kill cancer cells (Moazamian et al., 2018). Due to its important value in agriculture, environment and medicine, mechanisms of ICPs formation and sporulation in *B. thuringiensis* have been extensively investigated at the transcriptional level (Park et al., 1998; Zhang et al., 1998, 1999; Aronson, 2002; Doruk et al., 2013; Wanapaisan et al., 2013; Hui et al., 2014), as has the study of its post-transcriptional, post-translational, and metabolic pathways (Yousten and Rogoff, 1969; Glatron and Rapoport, 1972; Baum and Malvar, 1995; Mathy et al., 2007; Wang et al., 2013a; Zheng et al., 2015). Heretofore, there are no reports to describe if and how HSPs affect accumulation of ICPs in *B. thuringiensis*. Since, HSPs are so important for folding as the chaperons, we wanted to explore the effects that the absence of a single protein could have on the metabolism of *B. thuringiensis* and particularly on its ability to synthesize Cry proteins, one of the most abundant cellular proteins in bacterial cells.

Bt4.0718 is a highly virulent wide-type strain that was screened in our laboratory. It has been subjected to genome sequencing and shot gun proteomics at different stages of growth and sporulation (Huang et al., 2012; Jie et al., 2015). Our data have shown that Hsp20 (WP_001245065.1) protein can be detected in two processes of sporulation. Surprisingly, we found that the FPKM value of *hsp20* mRNA increases almost 10-fold from 63.04 to 702.98 from stages of early sporulation to late sporulation, as shown in Supplementary Figure S1. We therefore speculated that it is likely that Hsp20 may play an important role in sporulation and ICPs synthesis in Bt4.0718. In the present study, we deleted the *hsp20* gene from the genome and use the iTRAQ proteome (Liu et al., 2015; Wang S. et al., 2016; Zhang et al., 2018) to analyze the Hsp20 null strain. Our results demonstrated that deletion of Hsp20 affected expression of 195 DEPs and reduced synthesis of ICPs.

## Materials and Methods

### Strains and Culture Conditions

The strains and plasmids used in this study are listed in Table 1. *B. thuringiensis* strains were routinely cultured at 30°C and 120 rpm in fermentation medium, which was composed of 18 g/L glucose, 14.5 g/L tryptone, 2.5 g/L K_2_HPO_4_, 0.02 g/L FeSO_4_ ⋅ 7H_2_O, 0.02 g/L MnSO_4_ ⋅ H_2_O, and 0.25 g/L MgSO4 ⋅ 7H_2_O. Necessary antibiotics were added to the culture at the final concentrations of 50 μg/mL for kanamycin, 25 μg/mL for erythromycin, 300 μg/mL for spectinomycin or 60 units for polymyxin. *E. coli* DH5a and BL21(DE3) were used for cloning and expression of recombinant proteins, respectively. The *E. coli* strains were grown in Lysogeny broth (LB) medium or LB agar plates at 37°C. The antibiotic concentrations used were 50 μg/mL kanamycin, 100 μg/mL ampicillin and spectinomycin.

**TABLE 1:** Strains and plasmids.

**Strains and**		**References**
**plasmids**	**Characteristics**	**or sources**
Bt4.0718	Carrying the *Cry1Ac Cry2Aa* gene	Our lab
Bt4-*Δhsp20*	Bt4.0718 deleted the *hsp20* in the chromosome	This study
Bt4-*Δhsp20*::*hsp20*	The reverant	This study
*E. coli* DH5α	F^–^,*φ* 80d*lacZ* ΔM15, Δ(*lacZYA −argF*)U169, *deoR*, *recA1*, *endA1*, *hsdR17* (*rK*^–^, *mK*^+^), *phoA*, *supE44*, λ*^–^*, *thi −1*, *gyrA96*, *relA1*	TaKaRa
*E. coli* BL21(DE3)	Protein expression host; F^–^*dcm ompT hsdS* (rB^–^mB^–^) galλ(DE3)	Promega
pET28a (+)	T7 promoter expression vector, Km^R^	Novagen
pRP1028	*B. thuringiensis-E. coli* shuttle plasmid; Spc^R^, containing *turbo-rfp* gene and an I-SceI recognition site	JH^a^
pRP1028-*hsp20*UD	pRP1028 with the upstream and downstream regions of *hsp20*, intermediate vector in gene-knockout experiments	This study
pET28a-*hsp20*	*hsp20* in *Eco*RI and *Hin*dIII sites of pET28a, used for expression of Hsp20 in BL21(DE3)	This study
pSS4332	*B. thuringiensis-E. coli* shuttle plasmid; Km*^R^*, containing *gfp* and I-SceI restriction enzyme encoding gene	JH^a^
pSS1827	The helper plasmid for conjugative transfer; Amp^R^	JH^a^

*^*a*^State Key Laboratory of Agricultural Microbiology, College of Life Science and Technology, Huazhong Agricultural University, Wuhan, China.*

### DNA Manipulations

The whole genomic DNA of *B. thuringiensis* 4.0718 was extracted with rapid bacterial genomic DNA isolation kit (B518225, Sangon Biotech), and all steps carried out followed the standard manual. Primers used in the study are listed in Table 2. All polymerase chain reaction (PCR) products were purified using PCR product purification kit (B518141, Sangon Biotech). Recombinant plasmids were constructed in *E. coli* DH5α, and sequencing services were provided by Invitrogen.

**TABLE 2:** Primers used in this study.

		**Restriction**
**Primers**	**Sequence (5′–3′)**	**endonuclease**
P1 P2 P3 P4 P5 P6 16S-qF 16S-qR *odhA*-qF *odhA*-qR *dat*-qF *dat*-qR *leuA*-qF *leuA*-qR *mtnX*-qF *mtnX*-qR *acoA*-qF *acoA*-qR *murQ*-qF *murQ*-qR *tig*-qF *tig*-qR *tpx*-qF *tpx*-qR *eno*-qF *eno*-qR 1Ac-qF 1Ac-qR 1Aa-qF 1Aa-qR	TCCCCCGGG AACAGGAGAAGGAGAGAT TCGACGCGT AGAGAAGTTGTCGGATCAAG CGCGGATCC TTCTGGAACAAACTTAGGCG ATGCGTCGAC AATGGTTCAACAGCCTCATC CCGGAATTC CGTAATTTATTTCCAGAGATAACAACC CAAGCTT TTCGATATTAATTGTTGTCTTACTACTT AGAGTTTGATCCTGGCTCAG ACGGCTACCTTGTTACGACTT GACGAGGAAGAATACAACGACAA AAGCTCTTGTAATTCCGGATCA AGAGGTTCGCAGTTCGGAGAT GCTTTTGAGAAAGGGAGCGATA GCGAACAATCACCAGGAGTAAA AATGTCACTTTCTTTCGCTCTAGC ACCACCAGAAGCCGAAGAAGTA CCACTACGGATTTCAGCAGTTTCT ATCGTCCAGCGGAGCAAG TTGGCTACGTTTATATCCACCTG GAAAACAATGAATTTGGATGAGATG ACCAATGTAAATTAAACGTCCCTCT ATTAGAAGGTAACGTTGGCGTTT TCAATACCAAAGCGTTGTTCG TTAGAAACATACAAAGGCGAAGTG CGAATGGTAAATCAGCGCTAA GGTGAGCACGAAGCAGTAGAAT CCATAGATACACCAAGGATAGCG ATATTTCCTTGTCGCTAACGCA TGTACAAGAAATGCGTCCCATT ATCGCTCGTCTATCGGCATT CCAATGTTGATGGGAAACTACG	*Sma*I *Mlu*I *Bam*HI *Sal*I *Eco*RI *Hin*dIII

*The underline refers to restriction enzyme recognition sequence.*

### Construction of the Integrating Plasmids for Gene Deletion

To construct the plasmid for *hsp20* deletion, we amplified the upstream and downstream homologous arm of *hsp20*, approximately 700 bp from the *B. thuringiensis* 4.0718 genomic DNA by PCR using primer pairs of P1/P2, P3/P4. The upstream homologous arm was inserted into plasmid pRP1028 between the *Sma*I and *Mlu*I sites to construct the plasmid pRP1028-*hsp20*U. The downstream homologous arm was inserted into plasmid pRP1028-*hsp20*U between the *Bam*HI and *Sal*I sites to construct the integrating plasmid pRP1028-*hsp20*UD. pRP1028 is a shuttle plasmid with a temperature-sensitive suicide *B. thuringiensis* replicon. The resulting integrating plasmid was further verified by PCR and sequencing.

### Gene Knock-Out Procedure

The gene knock-out system in *B. thuringiensis* was developed based on homing endonuclease I-SceI mediated unmarked gene replacement method established for *B. anthracis* (Janes and Stibitz, 2006). To construct the *hsp20* deletion mutant Bt4-Δ*hsp20*, we included an I-SceI recognition site for I-SceI restriction endonuclease cleavage and one oriT site for conjugative transfer in pRP1028-*hsp20*UD. The vector also encodes RFP as a reporter protein and possesses a spectinomycin resistance marker for convenient screening. The subsequent procedures are illustrated in Supplementary Figure S2. The knockout strains and the complemented strains Bt4-Δ*hsp20*::*hsp20* were then screened. The related plasmids and strains were kindly provided by the Jin He of Huazhong Agricultural University.

### Heterologous Expression and Purification of Hsp20 in *E. coli* BL21(DE3)

The constructed plasmid pET28a-*hsp20* (use the primers P5/P6) was transformed into *E. coli* BL21(DE3). The overnight culture was diluted 100 times with fresh LB medium supplemented with 100 μgmL^–1^ kanamycin, then incubated and shaken at 37°C until the OD_600_ reached 0.6. Hsp20 expression was induced by adding isopropyl-β-d-thiogalactoside to a final concentration of 1 mM and incubated further for 4 h. The induced Hsp20 protein was purified by affinity chromatography in accordance with the protocol of HisTrap FF crude 1-mL column (GE Healthcare, Milwaukee, WI, United States), and was then used for primary anti-serum production in rabbit.

### Validation of *hsp20* Gene Deletion Strains

Two methods were used to identify whether *hsp20* was knocked out; one uses the primers P5/P6 for PCR identification and the other was Western blot (WB) detection. In the latter, 12 μg of proteins at 24 h were separated by NuPAGE^TM^ 4% to 12% Bis-Tris Gel (Invitrogen, United States), and was then blotted onto polyvinylidene difluoride membranes (Sigma) using a tank blot apparatus (Toyo, Tokyo, Japan), The transfer, blocking, hybridization, and washing steps were performed according to a previous study (Yang et al., 2015). The incubated primary rabbit antisera, at a dilution of 1:400, was labeled with anti-rabbit secondary antibody conjugated to goat DyLight 680 (Invitrogen, United States) at a dilution of 1:10000. The detection used the Odyssey infrared imaging system (Li-COR Biosciences, Lincoln, NE, United States).

### Growth Curve, Morphological Observation

The strains (OD_600_ = 0.6) were inoculated in approximately 1% in the fermentation medium (30 mL per 250 mL shake flask) and cultured at 30°C at 120 rpm. The growth curve was measured to 50 h and with three biological repetitions. The OD_600_ was measured to determine the growth rate of the cells by a spectrophotometer, and the samples were taken at each point in time to observe the difference between the strains.

### Preparation of Scanning Electron Microscope

Scanning Electron Microscopy of the 10-fold dilution of samples (mixture of spores and ICPs) were washed with sterile ultrapure water 10 times (9,000 × g, 3 min), and were then fixed with 2.5% glutaraldehyde for 12 h at 4°C. After washing three times with sterile ultrapure water, the samples were dehydrated gradually with 30, 50, 70, 80, 90, 95, and 100% ethanol, which was conducted twice for 10 min each. The samples were freeze dried and were then evenly applied on the cover glasses to sputtering gold plating. The samples were subsequently imaged with the scanning electron microscope (Hitachi Su8010, Japan).

### Sporulation Assays

Each strain was grown in the fermentation medium at 30°C for 60 h, and diluted to a final OD_600_ of 1.0. Cells were then heated to 65°C for 30 min, followed by gradient dilution (10×), and a series of 100 μL of diluents were coated onto LB plates. The colony-forming units (cfu) per mL were then counted.

### Extraction and Determination of ICPs Concentrations

For the extraction of the ICPs, the strains were grown under the GYS medium for 20 h, when cells started to lyse, 20 mL of each culture was separately collected. The procedures for extracting ICPs were performed according to previous studies (Wang X. et al., 2016; He et al., 2018). Finally, the ICPs were visualized by SDS-PAGE, and the ICPs concentrations were measured by the Bradford method.

### Virulence Assays

The larvae of *Heliothis armigera* were fed at 28°C with a light/dark cycle of 12 h. Artificial diet was comprised of 4 g yeast extraction, 7 g bean flour, 0.5 g vitamin C, 1.5 g agar, 36% acetic acid, and 1 g penicillin per 100 mL of water. The medium was transferred into 24-hole cell culture plates of approximately 1 mL per well (Corning, United States) with gradient concentration of the mixture of spores and crystals. Three strains were fermented for 48 h and suspended in distilled water after centrifugation. Finally, the semi-lethal concentration (LC_50_) were compared after a 48 h feeding of the SPSS (Inc., version 19.0, United States).

### Whole Cell Proteins Extraction

The harvested cells for the 18, 24, and 28 h were washed three times with PBS buffer and added with 300 μL lysis buffer, composed of 10 mL of cell lysate ratio of Urea 4.80 g, 4 mL 50 mM Tris–HCl (pH 8.0), Thiourea 1.52 g, NaCl 0.04 g, and CHAPs 0.40 g dissolved in 50 mM Tris–HCl, and placed with 10 μL protease inhibitor cocktail. The parameters of 3 s, 3 s, and 5 min were used to disrupt the cells by sonicated twice on ice (SONICS VCX150, United States). After cell disruption, the protein solution was separated by centrifugation (12000 × *g*, 10 min, 4°C). Protein concentration was determined using the Bradford protein assay kit (Thermo Fisher Scientific) and BSA as standard.

### iTRAQ Labeling

According to the iTRAQ protocol, 100 μg protein of each sample was digested with Trypsin Gold (Promega, United States) with the following ratio of protein: trypsin = 30:1 at 37°C for 16 h. Peptides were dried by vacuum centrifugation after trypsin digestion, and were then reconstituted in 0.5 M TEAB processed, according to the protocol for 8-plex iTRAQ reagent of the manufacture (AB SCIEX, United States). One unit of iTRAQ reagent was thawed and reconstituted in 24 μL isopropanol. Samples were labeled with the iTRAQ tags as follows: sample Bt4.0718 (119 tag) and *hsp20* knockout mutant (121 tag). Samples were tested in duplicates in a single run. The peptides were incubated with the isobaric tags, at room temperature for 2 h. The labeled peptide mixtures were then pooled and dried by vacuum centrifugation.

### LC-MS/MS Analysis

First dimensional separation at basic pH reverse phase – tryptic digestion mixture was dissolved in 100 μL mobile phase A (10 mM ammonium hydroxide in water/acetonitrile, 98/2 (v/v), pH = 10.5) and was then injected via 1200 Series HPLC (Agilent) into a Zorbax extend-C18 column (150 × 2.1 mm, Agilent). Mobile phase B was 10 mM ammonium hydroxide in water/acetonitrile, 10/90 (v/v), and the linear gradient elution was performed with 5%–50% B for 50 min at a flow rate of 0.3 ml/min. A total of 10 fractions from the first dimensional separation was collected (one fraction per 4 min) and then lyophilized for further second dimensional online LC-MS analysis.

Reverse-phase nano flow HPLC and tandem mass spectrometry – the second dimensional Chromatography was employed on the Eksigent nanoLC-Ultra^TM^ 2D System (AB Sciex, Concord, United States). The lyophilized SCX fractions were re-dissolved in 2% acetonitrile, 0.1% formic acid, and loaded on ChromXP C18 (3 μm, 120 Å) nano LC trap column. The online trapping and desalting procedure were carried out at 2 μL/min for 10 min with 100% solvent A. Solvents were composed of water/acetonitrile/formic acid (A, 98/2/0.1%; B, 2/98/0.1%). An elution gradient of 5%–38% acetonitrile (0.1% formic acid) in 70 min gradient was used on an analytical column (75 μm × 15 cm C18- 3 μm 120 Å, ChromXP Eksigent). LC MS/MS analysis was performed using a triple TOF 5600 System (AB Sciex, Concord, United States) fitted with nano spray III source. Data was acquired using an ion spray voltage of 2.4 kV, curtain gas of 30 PSI, nebulizer gas of 5 PSI, and an interface heater temperature of 150°C. The MS was operated with TOF-MS scans. For IDA, survey scans were acquired in 250 ms and as many as 30 product ion scans (80 ms) were collected if exceeding a threshold of 260 counts per second (counts/s), and with a + 2 to + 5 charge-state. A rolling collision energy setting was applied to all precursor ions for collision-induced dissociation. Dynamic exclusion was set 16 s.

Protein Identification and Quantification – The MS/MS data were analyzed for protein identification and quantification using protein pilot software v.4.5 (Sciex Inc., United States). The local false discovery rate was estimated with the integrated PSPEP tool in the protein pilot software to be approximately 1.0% and the tolerances were specified as ± 0.05 Da for peptides and ± 0.05 Da for MS/MS fragments. Searches were made against Bt DB27 protein database. The Bt DB27 database was read from the UniProt^1^, approximately 6284 sequences were searched, and parameters included iTRAQ 8-plex quantification, cysteine modified with IAA, trypsin digestion, thorough searching mode, and minimum protein threshold of 95% confidence (unused protein score >1.3). For a protein to be determined as differentially expressed the fold change must be ≥1.5.

### Bioinformatics Analysis

For a protein to be determined as differentially expressed the fold change must ≥1.5. The differentially expressed proteins (DEPs) were screened and then analyzed at the online website “OmicsBean,” the GO, and KEGG enrichment analysis were conducted. In the GO and KEGG pathway enrichment analysis of differentially expressed proteins, we compared the DEPs to all of the identified proteins as a background, and the formula used in these analyses was as follows: in which N is the number of GO/KEGG pathway entries in all identified proteins; n is the number of GO/KEGG pathway entries that represent differentially expressed proteins; M is the number of GO/KEGG pathway entries that can be matched to all identified proteins, and m is the number of GO/KEGG pathway entries that can be matched to a differentially expressed protein. If the *p*-value of the hypergeometric test was less than 0.05, the differentially expressed protein was significantly enriched in the GO/KEGG pathway entry.

$$\text{P}=1-\sum_{i=0}^{m-1}\frac{\left(\begin{array}{c} M\\ i \end{array}\right)\,\left(\begin{array}{c} N-M\\ n-i \end{array}\right)}{\left(\begin{array}{c} N\\ n \end{array}\right)}$$

### RNA Extraction and Quantitative Real-Time PCR (qRT-PCR)

Total RNA was extracted from the bacterial cells harvested at the time point of 28 h. Bt4-Δ*hsp20* and Bt4.0718 were cultivated using Trizol Reagent (15596-026 Invitrogen Biotech). Following digestion of the residual genomic DNA by DNase I (EN0521, Thermo Fisher Scientific), 1 μg of total RNA was reverse transcribed to cDNA utilizing RevertAid^TM^ First Strand cDNA Synthesis Kit (12328040, Thermo Fisher Scientific) according to the instruction of the manufacturer. The two-step real-time RT-PCR was conducted using power SYBR^®^ green PCR master mix (4367659, Thermo Fisher Scientific) in a CFX connect Real-Time PCR detection system (Bio-Rad). The primers used in this study are summarized in Table 2. 16S rRNA was used as a normalization control for gene expression.

## Results

### Validation of *hsp20* Knock-Out and Complemented Strain

To determine the role of Hsp20 in sporulation and ICPs production, we constructed an *hsp20* mutant. PCR and WB analyses were performed to confirm the positive clones. First, the upstream and downstream homology arms of *hsp20* were confirmed to be ligated to the pRP1028 vector after sequencing. The positive clones of the knockout (Bt4-Δ*hsp20*) and complemented strain (Bt4-Δ*hsp20*::*hsp20*) were identified by PCR (16S rRNA was used as the blank control). The expression of pET28a-*hsp20* was then induced in BL21(DE3) to prepare the polyclonal antibody, and whole proteins of the three strains were extracted for WB analysis verification (30S ribosome protein served as the internal reference). The final results revealed that the knockout and complemented mutations of *hsp20* in Bt4.0718 were successful (Supplementary Figure S3).

### Growth Curve Comparison of the *hsp20* Knockout Strain and Bt4.0718

**FIGURE 1:**
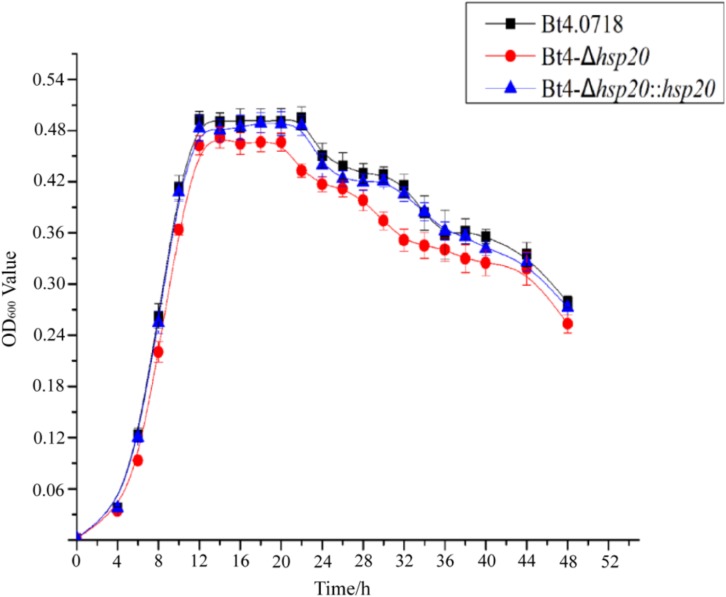
Growth of the *hsp20* mutant shows a marked decay at end of stationary phase. Growth curves of the Bt4.0718, Bt4-Δ*hsp20*, and Bt4-Δ*hsp20*::*hsp20* strains which a bar for each sampling time point represents the standard error of the mean from three batches.

By comparing the growth curves of Bt4.0718, Bt4-Δ*hsp20*, and Bt4-Δ*hsp20*::*hsp20*, we found that the stability period of Bt4-Δ*hsp20* was reduced by nearly 2 h and that the maximum biomass was also slightly reduced. The OD_600_ of Bt4.0718 peaked at 16.48, and the stationary phase ended after 22 h, which was only slightly different from that of Bt4-Δ*hsp20*::*hsp20*. The highest OD_600_ of Bt4-Δ*hsp20* was 14.43 and entered the decline phase at 20 h (Figure 1).

### Effects of *hsp20* Deletion on the Sporulation and ICPs

**FIGURE 2:**
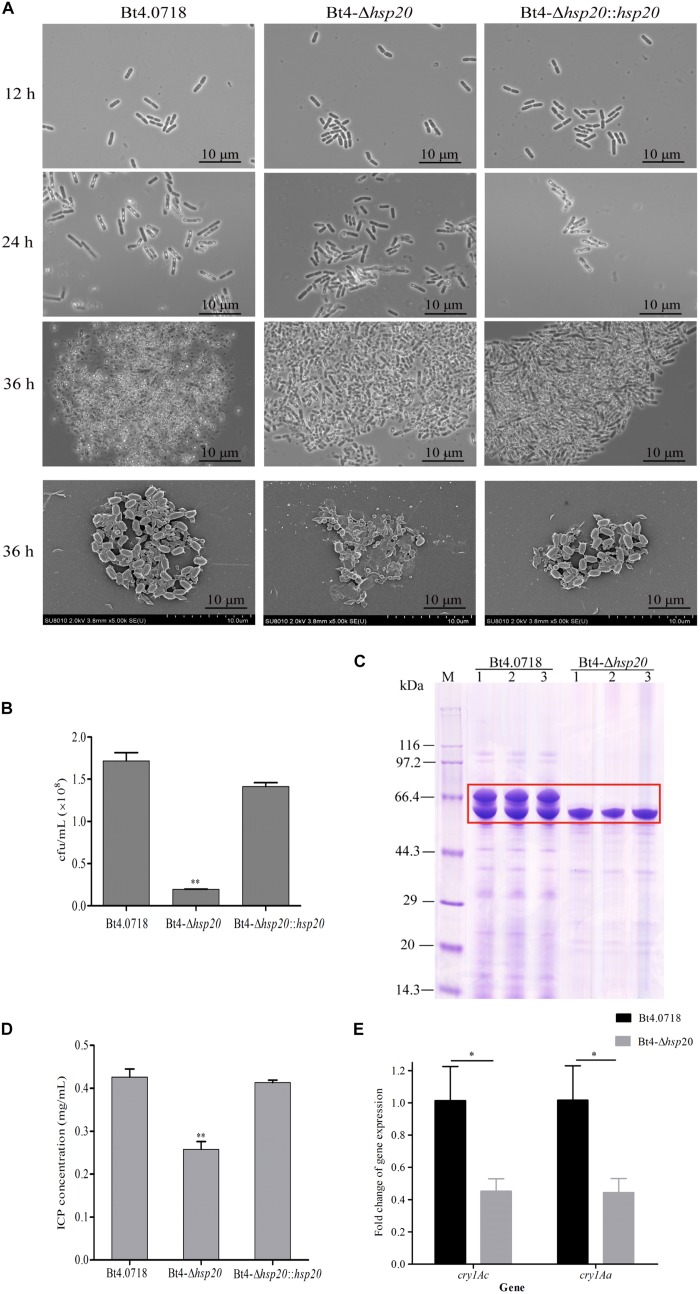
Sporulation and ICPs formation are reduced in Bt4-Δ*hsp20*. **(A)** Phase contrast microscopy (100 × oil objective) of the strains at 12, 24, and 36 h; scanning electron microscopy of the spores and crystal proteins (10.0 K×) at 36 h. **(B)** Sporulation of the Bt4-Δ*hsp20* compared to Bt4.0718, and Bt4-Δ*hsp20*::*hsp20* strains in LB at 60 h. Spore numbers per mL were indicated by cfu/mL, and each experiment was carried out in triplicate which the values were means ± standard deviations. Significances of differences by Student’s *t*-test are indicated (^∗∗^*P* ≤ 0.01). **(C)** ICPs in Bt4.0718 and Bt4-Δ*hsp20* strains at 20 h analyzed by SDS-PAGE, ICPs were boxed in red. **(D)** Concentrations of ICPs at 20 h determined by Bradford method in Bt4-Δ*hsp20* compared to Bt4.0718, and Bt4-Δ*hsp20*::*hsp20* strains. The values were means ± standard deviations for triplicate assays, and the significances of differences by Student’s *t*-test is indicated (^∗∗^*P* ≤ 0.01). **(E)** qRT-PCR analysis the fold-change of *cry1Ac* and *cry1Aa* in Bt4-Δ*hsp20* strain which compared to Bt4.0718 after fermentation for 28 h. The values were means ± standard deviations for triplicate assays, the significances of differences by Student’s *t*-test are indicated (^∗^*P* ≤ 0.05).

The results of phase contrast microscopy revealed no difference in the phenotypes of the three strains at 12, 24, and 36 h, and only a very small number of spores were produced in Bt4-Δ*hsp20* compared with that of Bt4.0718 and Bt4-Δ*hsp20*::*hsp20*. Bt4-Δ*hsp20* generated few immature spores, whereas Bt4.0718 released a large number of mature spores and obvious rhombic parasporal crystal proteins. A mass of mature spores was also observed in the Bt4-Δ*hsp20*::*hsp20* cells at 24 and 36 h. Subsequently, the mixture of spores and crystals of the three strains was observed under a scanning electron microscope (Figure 2A). Aside from the cell and crystals density, no significant difference was observed in the shape and size of the spores and crystal proteins produced by the three strains.

Sporulation assays of each strain revealed that Bt4.0718 and the complemented strain produced 1.72 × 10^8^ and 1.41 × 10^8^ cfu/mL, respectively. In contrast, the *hsp20* knockout strain produced 1.94 × 10^7^ cfu/mL, which is only 11.8% of that observed in Bt4.0718 (Figure 2B). This result indicated that *hsp20* was important in the development and generation of spores. The deletion of *hsp20* may lead to the reduction in ICPs yield, as observed in Figure 2C. To be quantitative, we measured the concentration of ICPs, and conducted the qRT-PCR of *cry1Ac* and *cry1Aa*, which are the main toxin genes in the Bt4.0718 stain as shown in Figures 2D,E. The concentration of ICPs in Bt4.0718 was 0.426 μg/mL, which was 1.65 times greater than that of knockout strain (0.257 μg/mL), and the complemented strain was 0.413 μg/mL. Whereas, the expression revealed by the qRT-PCR of *cry1Ac* and *cry1Aa* in *hsp20* knockout strain was nearly half of that exhibited by the Bt4.0718. Due to the quantitative results of iTRAQ on Cry1Ac and Cry1Aa, which are specific proteins of *B. thuringiensis* with some limitation, the peptides peak area output was calculated as shown in Supplementary Figure S4 (according to the Supplementary Table S1 which from the mass spectrometry proteomics results), and the area level of Cry1Ac and Cry1Aa in Bt4.0718 were approximately 1.623 times that of *hsp20* knockout strain (SE = 0.210, ^∗^*P* < 0.05).

Virulence assays of the larvae of *H. armigera* showed that the LC_50_ of the *hsp20* knockout strain was 10.789 μg/mL, whereas the toxicity of Bt4.0718 and the complemented strain were 5.254 and 5.613 μg/mL, respectively (Table 3). This result was consistent with the quantification of ICP.

**TABLE 3:** Virulence assays of the larvae of *Heliothis armigera*.

**Strain**	**LC_50_ (μg/mL)**	**95% confidence interval**
Bt4.0718	5.254	4.356–6.352
Bt4-Δ*hsp20*	10.789	8.869–13.752
Bt4-Δ*hsp20*::*hsp20*	5.613	4.610–6.879

### SDS-PAGE Analysis of Whole Cell Proteins of Bt4.0718 and Bt4-Δ*hsp20*

To detect differences at the proteome level, we carried out the SDS-PAGE analysis of whole proteins of Bt4.0718 and Bt4*-*Δ*hsp20* at three time points (18, 24, and 28 h), as shown in Supplementary Figure S5. The protein difference at the T3 period (28 h) increased, and it was selected to run the comparative quantitative proteomic analyses.

### Analysis of Differentially Expressed Proteins (DEPs) Between the Bt4.0718 Strain and the *hsp20* Knockout Mutant

**FIGURE 3:**
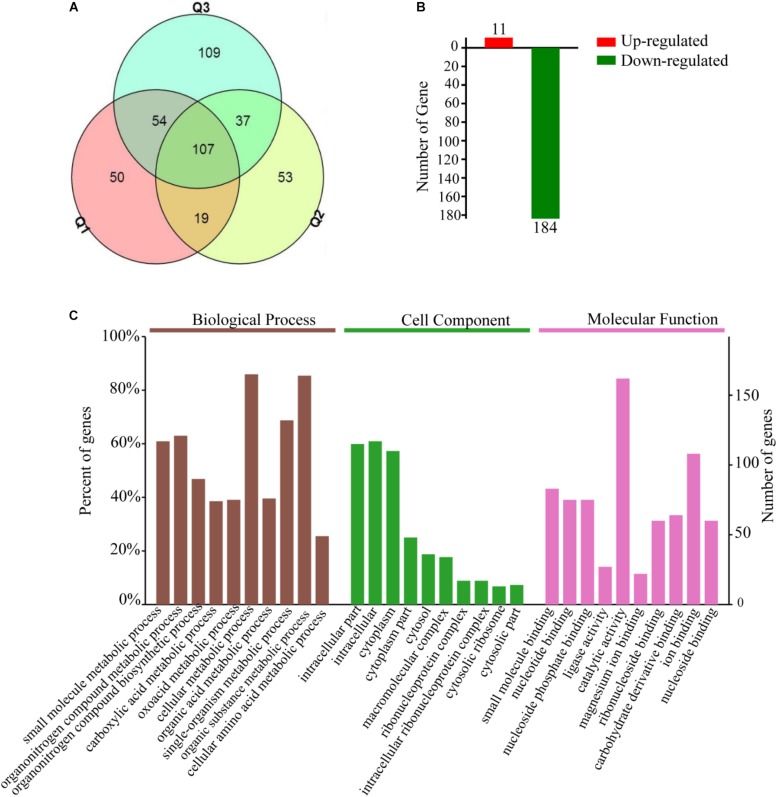
Total of 195 differential expression proteins are screened which Bt4-Δ*hsp20* compared to Bt4.0718, and most of them are main involved in intracellular substance and cytoplasm metabolism. **(A)** Venn diagram of differential proteins from every biological repetition (Q1, Q2, and Q3). **(B)** Number of screened up or downregulated differential expression proteins (11 and 184 were upregulated and downregulated, respectively). **(C)** GO annotations of the 195 DEPs. Biological processes, cellular component, and molecular function in the three categories significantly enriched and analyzed that ranked the top ten. The entries for each category were arranged according to their *p*-value size from left to right in order of priority. Based on the vertical axis, each entry can be viewed corresponding to the percentage of total differences in protein expression. A Go term with a *p*-value of <0.05 was considered significant.

A total of 1736, 1713, and 1769 proteins were identified in three biological replications (Supplementary Table S2, from the mass spectrometry proteomics results). A fold change ≥1.5 was considered to be differentially expressed. In addition, reproducibility analysis by a Venn diagram showed that the differential proteins shared by three and two groups accounted for 60, 50, and 42% of the total number of differential proteins in groups Q1, Q2, and Q3 with good repeatability, respectively (Figure 3A). A total of 195 DEPs were then identified; approximately 11 were upregulated and 184 were downregulated (Figure 3B), and in the GO and KEGG analysis.

The DEPs were subjected to GO enrichment analysis and were classified as biological process, molecular function or cellular component. The three main biological processes are cellular metabolism, organic substance metabolism, and single-organism metabolism. The molecular function is mainly distributed in catalytic activity and ion binding. The main cellular components are intracellular substance and cytoplasm (Figure 3C).

### KEGG Analysis of DEPs

**FIGURE 4:**
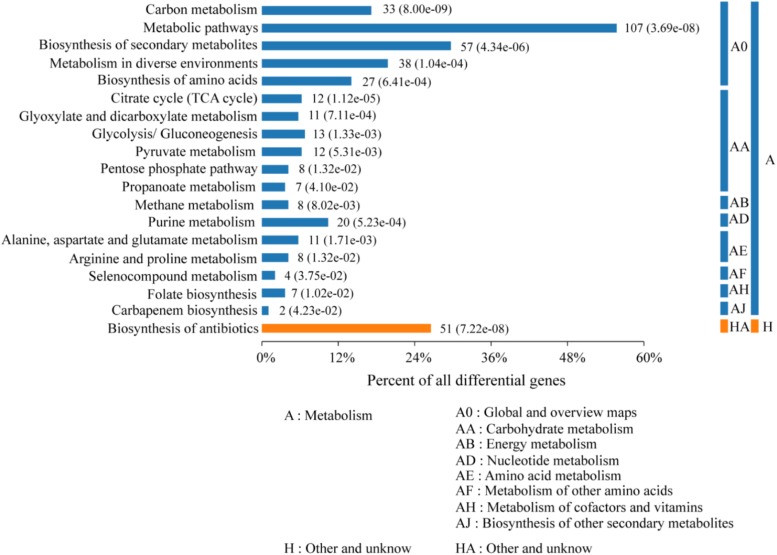
Bar chart of enriched KEGG pathways of the 195 differential expression proteins. The number of involved proteins in a specific pathway and the corresponding *p*-value are shown on the right side of the column.

According to the KEGG categories, the proteins were annotated into 18 groups (Figure 4). Carbon metabolism, metabolic pathways, biosynthesis of secondary metabolites, microbial metabolism in diverse environment, and biosynthesis of amino acid were classified as the global and overview maps in metabolism. Whereas, energy, nucleotide, amino acid, cofactors, and vitamins metabolism dominated the cellular or single-organism metabolic processing. Nearly all upregulated genes, such as *upp*, *acoA*, *dat*, *hom*, *leuA*, *mtnX*, *valS*, and *rplM*, were classified under energy citric acid cycle (TCA) and amino acid metabolism, including D-alanine, leucine, proline, lysine, cysteine, methionine, and so on, and only *sigA* and *spoIIAB* were related to sporulation.

### Validation of DEPs by qRT-PCR

**FIGURE 5:**
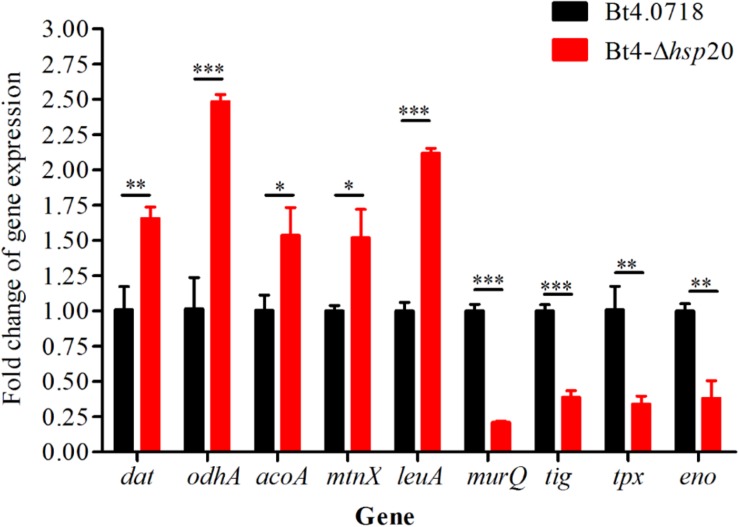
Validation of the differential expression of nine genes. Gene expression level by qRT-PCR of Bt4-Δ*hsp20* compared with Bt4.0718. The values were means ± standard deviations for triplicate assays, the significances of differences by Student’s *t*-test are indicated (^∗^*P* ≤ 0.05, ^∗∗^*P* ≤ 0.01, ^∗∗∗^*P* ≤ 0.001).

The proteomic results were verified at the mRNA level. For this purpose, we carried out qRT-PCR quantification of eight genes, including four upregulated genes, namely, *dat*, *odhA*, *mtnX*, and *leuA* (fold change were 1.66, 2.48, 1.52, and 2.12, respectively), and four downregulated genes, namely, *murQ*, *tig*, *tpx*, and *eno* (fold change were 0.21, 0.39, 0.34, and 0.38, respectively). The *acoA* was, additively, 1.54-fold for qRT-PCR quantification. The results showed that the eight proteins exhibited a similar change trend both at the mRNA level and protein level (Figure 5).

## Discussion

### The Possible Effect of Hsp20 in Early Sporulation

Phase contrast microscopy results suggested that *hsp20* influenced early sporulation. Six proteins that are directly related to the sporulation exhibited differential expression in the mutant (two upregulated and four downregulated). SpoIIAB and SigA are upregulated 1.54-fold and 1.56-fold, respectively. SpoIIAB is known to have sigma factor antagonist activity that binds and reduces or prevents the rate sigmaF factor transcription. SigmaF factor subsequently governs the transcription of *spoIIIG*, which encodes the late prespore-specific regulator sigmaG (Serrano et al., 2004). In Bt4.0718 strain, SigA remains constant during the transition from vegetative growth to sporulation. The sporulation-specific sigma factors appear successively, in the order of SigmaH, SigmaE, and SigmaK (Fujita, 2000), and are overexpressed in sporulation mutants of *B. thuringiensis.* These mutants are blocked in the phosphorylation of Spo0A, a key regulator of sporulation initiation (Baum and Malvar, 1995). Whereas, the promoters of a number of cry proteins can be driven by SigmaE and SigmaK (Zhang et al., 1998), and *cry* genes, including *cry1*, *cry4*, *cry8*, *cry11*, and *cry18*, which are controlled by both SigE and SigK (Deng et al., 2014). Thus, this may be the reason for the obvious difference in the impact degree of sporulation and ICP formation.

On the other hand, of the downregulated proteins (RacX, SpovG, FtsA, and FtsH), FtsA and FtsH are related to cell division, sporulation and are involved in switching the site of cell division from the mid-cell to polar position (Kemp et al., 2002). FtsA is under the control of SigmaF, whereas FtsH is an ATP-dependent zinc metallopeptidase for both cytoplasmic and membrane proteins. FtsH is initially asymmetrically located in the septa of the sporulation cells and subsequently in the membrane that engulfed the fore-spore during sporulation (Wehrl et al., 2000). Spo0A is important in initiating sporulation; the amount of the master regulator protein Spo0A is reduced in an *ftsH* knockout and the small amounts of Spo0A protein present are inactive (Ai and Schumann, 2009). Whereas, RacX exhibits an amino acid racemase activity and is probably involved in peptidoglycan modification during cortex synthesis for sporulation from the III to IV stage. *spoVG* controls the sporulation at the V stage, leading to an asymmetric septation and causing the accumulation of ICPs (Rosenbluh et al., 1981; Crissman et al., 1989; Matsuno and Sonenshein, 1999).

### Possible Influences of *hsp20* Knockout on Amino Acid Supply at Stationary Phase

During sporulation, some operons and genes involved in amino acid biosynthesis and transport were upregulated in response to amino acid starvation (Wang et al., 2013b). However, among the DEPs, twelve 50S ribosomal protein subunits (*rplA/C/D/E*, etc.) required for the assembly of 50S ribosomal protein and formation of peptide bond (Nissen et al., 2000) are down-regulated, (0.1 to 0.61-fold) thus the translation level may be decreased. As well as that, 13 kinds of tRNA ligase, which include *alaS*, *argS*, *asnS*, *valS*, *proS*, *metG*, etc., are responsible for the corresponding amino acid transfer in the translation process. Meanwhile, 13 genes involved in the biosynthesis of 9 kinds amino acids, such as *proA/B/S*, participate in the proline production from glutamate. GlnA catalyzes glutamate into glutamine; *hisA/D* promote histidine formation; *metD/E*, *argD*, *glyA*, and *dapB/H* are involved in the syntheses of methionine, arginine, glycine, and lysine, respectively, all of which are also down-regulated. These aspects reflect the shortage of amino acids.

In fact, 80% of amino acids for ICP synthesis mainly came from protein turnover (Monro, 1961), ATP-dependent protease, usually with high expression, could quickly degrade some abnormal polypeptides (Frees et al., 2007; Molière and Turgay, 2009) to provide enough amino acids during sporulation. ATP-dependent protease ClpX, HslU/HslV was observed without significant differences. But, two peptidases observed from the DEPs are down-regulated (0.40-fold, 0.44-fold), which include the cytosol aminopeptidase (PepA), the enzyme CtpA with endopeptidase activity. Therefore, *hsp20* could likely contribute to maintain a normal supply of amino acids during sporulation and ICP formation during amino acid starvation.

### Energy and Carbon Source Could Be Impacted by Hsp20 During Sporulation

Energy is essential to any microbial life activity, and according to the KEGG analysis of DEPs, some downregulated proteins are involved in TCA cycle and glycolysis, which are required to provide the main ATP for the Bt cells at the stationary phase. The abundant glucose of exponential growth phase mainly flows into glycolysis pathway to produce pyruvate, which can be utilized by other pathways, and the first three enzymes of TCA cycle are critical for sporulation (Jin and Sonenshein, 1994; Ireton et al., 1995). CitZ and CitB participate in the synthesis of citrate. CitZ is involved in the first step of the synthesis of isocitrate from oxaloacetate. CitB catalyzes the reversible isomerization of citrate to isocitrate via *cis-*aconitate to produce acetyl-coA, which is linked to fatty acid metabolism. If a loss in activity results in sporulation defect, they can be regulated by phosphorylated Spo0A, which is involved in the regulation of citrate concentration and in sporulation (Craig et al., 1997). Furthermore, aconitase (encode by *citB*) is required for efficient late-sporulation gene expression: its RNA binding activity may stabilize *gerE* mRNA for proper timing of spore coat assembly (Serio et al., 2006).

In additional, Eno, Pyk, and GpmI are downregulated in the subpathway that catalyzes the reversible conversion of 2-phosphoglycerate into phosphoenolpyruvate. This subpathway is essential for the degradation of carbohydrates via glycolysis. Eno interacts with GroEL, which is involved in sporulation (Endo and Kurusu, 2007). A reduction in the expression of PdhC will decrease the conversion of pyruvate into acetyl-CoA that is involved in glycolysis, ultimately reducing the energy. As a component of the 2-oxoglutarate dehydrogenase complex, down regulated OdhB will reduce the overall conversion of 2-oxoglutarate to succinyl-CoA. The two down regulated proteins, SucD, and SucC are succinate-CoA ligase (ADP-forming) alpha and beta subunits, which are involved in the subpathway that synthesizes succinate from succinyl-CoA and the TCA, which couples the hydrolysis of succinyl-CoA to the synthesis of either ATP or GTP.

For the carbon source, the poly-3-hydroxybutyrate (PHB) and acetoin are commonly produced and excreted during exponential phase as the intercellular and extracellular carbon and energy store in *B. thuringiensis*. However, PHB metabolism has been indicated to be unrelated to sporulation and parasporal crystal protein formation (Wang X. et al., 2016), and no related DEPs were observed. Moreover, we observe that *acoA* is upregulated approximately 1.54-fold (only identified with qRT-PCR). This protein mainly catalyzes the 2,6-dichlorophenolindophenol cleavage of acetoin into acetate and acetaldehyde to utilize acetoin as the carbon source for growth (Huang et al., 1999), serving as an alternative to energy supplementation and self-rescue. Hence, acetoin could also be important as the carbon and energy source for sporulation and ICP formation during progressive deficiency of nutrients at stationary phase.

### Hsp20 May Cause Some Effects on Purine Metabolism, mRNA Stability During Sporulation and ICP Formation

Down-regulated proteins: PurA/B, adenylate kinase (Adk), and nucleoside diphosphate kinase (Ndk), could diminish purine metabolism. The *purA/B/M/N/S* code enzymes are involved in both IMP and AMP biosynthesis, which are part of purine biosynthesis (Saxild and Nygaard, 1988; Mäntsälä and Zalkin, 1992). Ndk is involved in the synthesis of nucleoside triphosphates other than ATP. Adk catalyzes the reversible transfer of the terminal phosphate group between ATP and AMP.

Downregulated RpoB and RpoE are DNA-directed RNA polymerase subunits that catalyze the transcription of DNA into RNA using the four ribonucleoside triphosphates as substrates. Moreover, these proteins participate in both the initiation and recycling phases of transcription and are involved in the sigma factor switching that initiates sporulation (Moeller et al., 2012).

For the mRNA stability, two correlated downregulated DEPs were found, namely polyribonucleotide nucleotidyltransferase (Pnp) and ribonuclease J1 (RnjA). Pnp is necessary for competence development in *Bacillus subtilis* but exerts no effect on sporulation efficiency. By contrast, RnjA exhibits endonuclease and 5′–3′ exonuclease activities, and is involved in both rRNA and mRNA stability and degradation, and its knockout mutant causes major defects in sporulation (Figaro et al., 2013).

**FIGURE 6:**
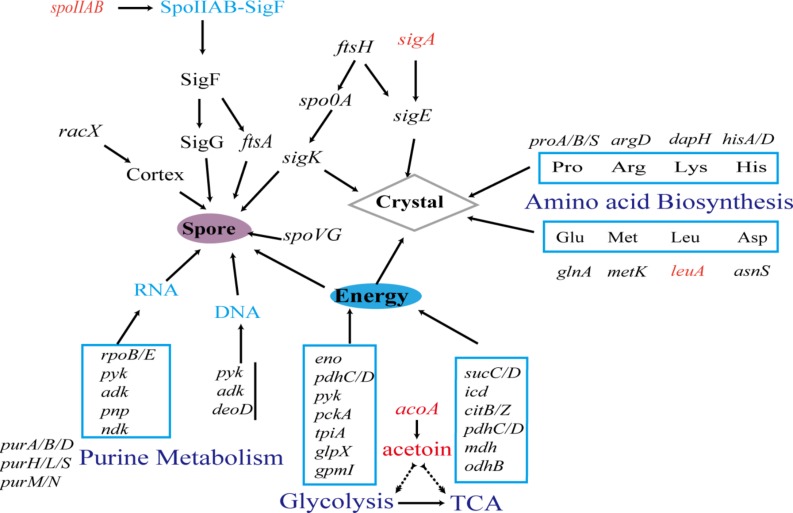
Simplified diagram of the effects of *hsp20* knockout on the sporulation and ICPs. The genes, proteins, and amino acids are included in the diagram. Black and red indicate downregulation or decrease and upregulation or increase, respectively.

Figure 6 shows the pathways by which the DEPs (such as the Table 4) observed in the *hsp20* deletion could negatively affect sporulation and ICPs formation. Hsp20 may function in aiding protein folding or aiding translation of these proteins under the starvation conditions leading to sporulation and ICP formation.

**TABLE 4:** Part of differentially expressed proteins involved in discussion.

	**Fold**	**Gene**	
**Accession**	**change**	**name**	**Protein**
W8Y895	2.88	*odhA*	2-oxoglutarate dehydrogenase E1 component
W8Y7T1	2.63	*mtnX*	2-hydroxy-3-keto-5-methylthiopentenyl-1-phosphate phosphatase
W8Y091	2.19	*leuA*	2-isopropylmalate synthase
W8YKG0	1.70	*upp*	Uracil phosphoribosyltransferase
W8YBF1	1.65	*dat*	D-alanine aminotransferase
W8Y805	1.54	*spoIIAB*	Anti-sigma F factor
W8YGG2	1.56	*sigA*	RNA polymerase sigma factor SigA
W8YJX1	0.62	*argS*	Arginine – tRNA ligase
W8Y7Y7	0.61	*hisA*	imidazole-4-carboxamide isomerase
W8YF62	0.59	*proS*	Proline – tRNA ligase
W8YJT8	0.57	*gpmI*	2,3-bisphosphoglycerate-independent phosphoglycerate mutase
W8YA17	0.56	*pckA*	Phosphoenolpyruvate carboxykinase (ATP)
W8XX55	0.55	*purB*	Adenylosuccinate lyase
W8YG53	0.55	*ftsA*	Cell division protein FtsA
W8Y765	0.55	*metK*	*S*-adenosyl methionine synthase
W8Z6B2	0.54	*citZ*	Citrate synthase 2
W8Y9L9	0.54	*pyk*	Pyruvate kinase
W8YFQ3	0.54	*rnjA*	Ribonuclease J1
W8Y5K4	0.53	*purH*	Bifunctional purine biosynthesis protein PurH
W8Z3Q1	0.51	*pnp*	Polyribonucleotide nucleotidyltransferase
W8Y9K4	0.47	*mdh*	Malate dehydrogenase
W8Y6I8	0.46	*citB*	Aconitate hydratase A
W8Y5Y6	0.46	*thiM*	Hydroxyethylthiazole kinase
W8Y9G2	0.46	*icd*	Isocitrate dehydrogenase [NADP]
W8Y7A6	0.46	*sucD*	Succinate – CoA ligase [ADP-forming] subunit alpha
W8YK96	0.45	*metG*	Methionine – tRNA ligase
W8Y8A7	0.45	*ndk*	Nucleoside diphosphate kinase
W8Z497	0.44	*pdhC*	Dihydrolipoyllysine-residue acetyltransferase
W8YKA7	0.44	*mfd*	Transcription-repair-coupling factor
W8YSS4	0.43	*purN*	Phosphoribosylglycinamide formyltransferase
W8Y8Z7	0.43	*argD*	Acetylornithine aminotransferase
W8YC93	0.41	*ftsH*	ATP-dependent zinc metalloprotease
W8YKA3	0.40	*spoVG*	Putative septation protein SpoVG
W8XWN5	0.39	*adk*	Adenylate kinase
W8YC55	0.39	*purA*	Adenylosuccinate synthetase
W8Z3C3	0.35	*glnA*	Glutamine synthetase
W8YDI9	0.32	*proB*	Glutamate 5-kinase 1
W8YWA0	0.31	*deoD*	Purine nucleoside phosphorylase DeoD-type
W8Z185	0.30	*proA*	Gamma-glutamyl phosphate reductase
W8Z4I3	0.28	*rpoB*	DNA-directed RNA polymerase subunit beta
W8Z819	0.26	*eno*	Enolase
W8Y537	0.49	*rplA*	50S ribosomal protein L1
W8YSF0	0.42	*rplE*	50S ribosomal protein L5
W8YHI6	0.39	*alaS*	Alanine – tRNA ligase
W8Y9C4	0.57	*asnS*	Asparagine – tRNA ligase
W8YJP5	0.51	*glyA*	Serine hydroxymethyltransferase
W8YIL1	0.40	*pepA*	cytosol aminopeptidase
W8Y592	0.44	*ctpA*	Carboxy-terminal processing protease

*These genes selected according to the metabolism pathway would influence the sporulation and insecticidal crystal protein formation.*

## Conclusion

The *hsp20* would influence the production of sporulation and ICPs, which are the hallmarks of Bt, and the general metabolism. Stationary growth phase of the Bt4-Δ*hsp20* was reduced by approximately 2 h, and only a slight decrease was observed in the biomass. By analyzing the DEPs of the iTRAQ proteomics Bt4-Δ*hsp20* compared to Bt4.0718, it was evident that the deletion of *hsp20* caused downregulation of a lot of proteins, which are distributed in multiple metabolic pathways. This most likely cause the decrease in 50S ribosome assembly and its translation level, the short supply of amino acids (the biosynthesis and the protein-turnover), the drop in purine metabolism and some mRNAs stability, the insufficient energy supply (including TCA, glycolysis) during sporulation and ICP formation at the stable phase.

## Ethics Statement

This study was approved by the Ethics Committee and Animal Care and Use Committee of Hunan Normal University, and all animals were handled in accordance with the guidelines of Hunan Provincial Council on Animal Care.

## Author Contributions

XD and LX conceived and designed all the experiments. JX and JP conducted the proteomic studies (iTRAQ) and drafted the manuscript. TZ, ZY, and XZ performed the qRT-PCR analysis. MQ and SY participated in the Western blot analysis. SL and JL analyzed the proteomic data. PZ contributed to the bacterial culture experiments. XD provided reagents, materials, and analysis tools.

## Conflict of Interest Statement

The authors declare that the research was conducted in the absence of any commercial or financial relationships that could be construed as a potential conflict of interest.

## Footnotes

1 http://www.UniProt.org/uniprot/

## Supplementary Material

FIGURE S1 Line graph of *hsp20* mRNA FPKM value at early sporulation and late sporulation (the bar of two points represents the standard error of mean).

FIGURE S2 Schematic of the I-SceI mediated marker-less gene knockout method.

FIGURE S3 Identification of *hsp20* knockout. **(A)** Lanes 1, 2: knockout vector pRP1028-*hsp20*UD. **(B)** Colony PCR amplifies the *hsp20* gene with P5/P6. Lane 1: wide type strain; Lane 2: complemented strain; Lanes 3, 4: knockout strain; Lanes 5, 6: 16S rRNA sequence of Bt4.0718 and the *hsp20* knockout strain. **(C)** Western blot analysis of *hsp20* expression in the three strains. Lane 1: complemented strain; Lane 2: Bt4.0718 strain; Lane 3: *hsp20* knockout strain.

FIGURE S4 Peptides peak area of Cry1Ac and Cry1Aa in Bt4.0718 and *hsp20* knockout strain (Std. Error = 0.210, ^∗^*P* < 0.05).

FIGURE S5 SDS-PAGE of the whole cell protein samples of Bt4.0718 and the *hsp20* knockout strain at different time (lanes 1–3: 18, 24, and 28 h of Bt4.0718 strain; lanes 4–6: 18, 24, and 28 h of the *hsp20* knockout strain).

TABLE S1 The quantitative ICP peptides results.

TABLE S2 The quantitative proteome results.
