# Supplementary figures and images for: Role of hsp20 in the Production of Spores and Insecticidal Crystal Proteins in Bacillus thuringiensis
Source: Front Microbiol. 2019 Sep 4;10:2059. doi: 10.3389/fmicb.2019.02059 (PMC6737285; doi:10.3389/fmicb.2019.02059)

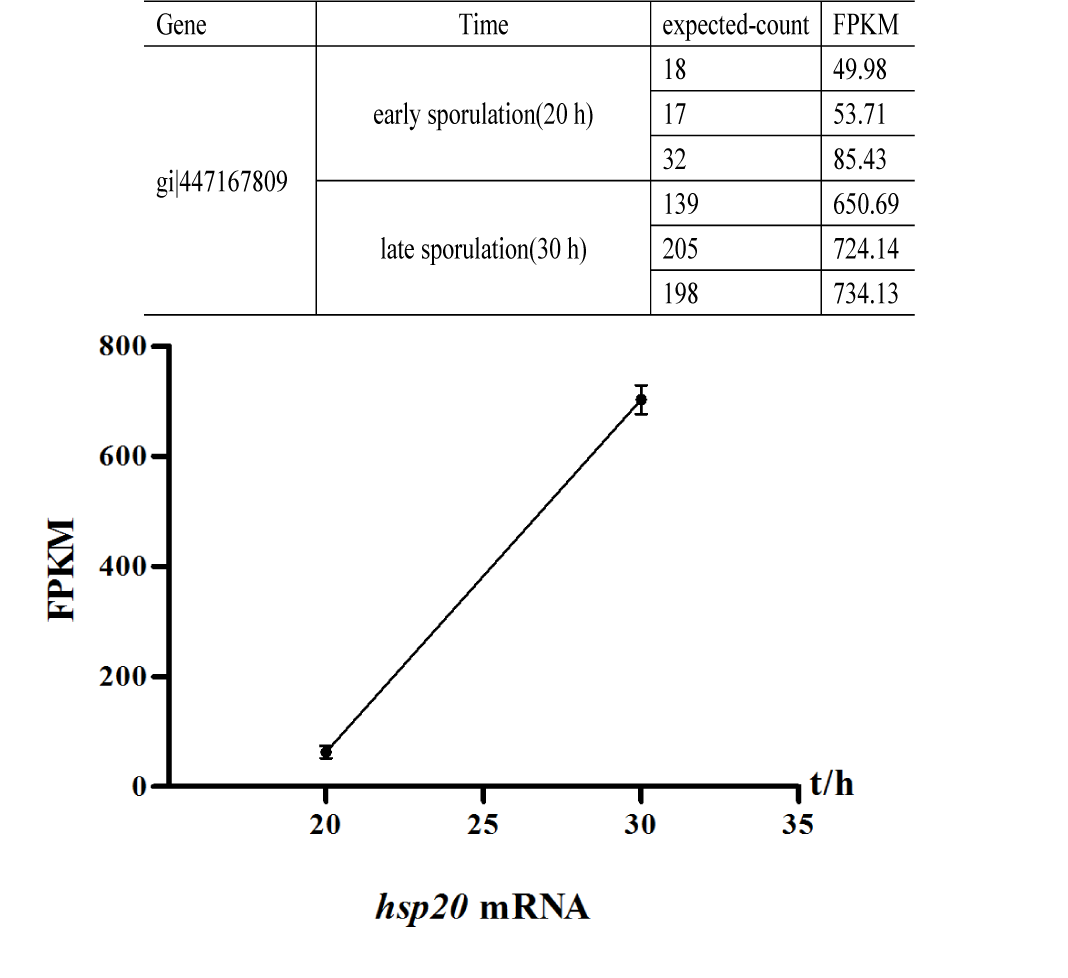

Supplement: FIGURE S1 — Line graph of hsp20 mRNA FPKM value at early sporulation and late sporulation (the bar of two points represents the standard error of mean). [file Image_1.TIF]

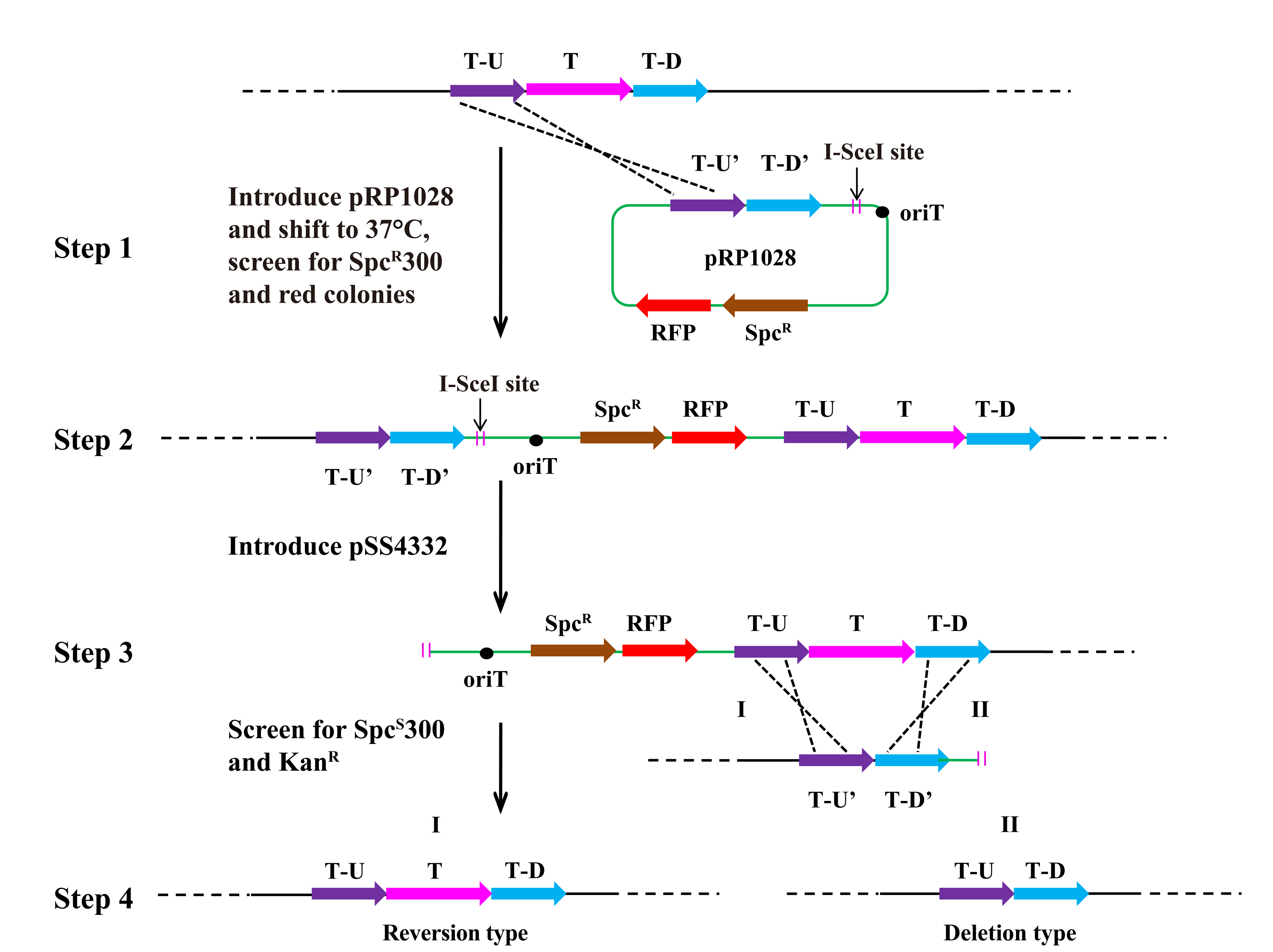

Supplement: FIGURE S2 — Schematic of the I-SceI mediated marker-less gene knockout method. [file Image_2.TIF]

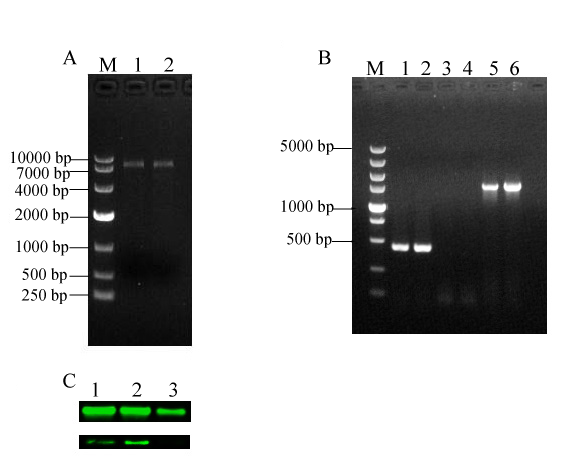

Supplement: FIGURE S3 — Identification of hsp20 knockout. (A) Lanes 1, 2: knockout vector pRP1028-hsp20UD. (B) Colony PCR amplifies the hsp20 gene with P5/P6. Lane 1: wide type strain; Lane 2: complemented strain; Lanes 3, 4: knockout strain; Lanes 5, 6: 16S rRNA sequence of Bt4.0718 and the hsp20 knockout strain. (C) Western blot analysis of hsp20 expression in the three strains. Lane 1: complemented strain; Lane 2: Bt4.0718 strain; Lane 3: hsp20 knockout strain. [file Image_3.TIF]

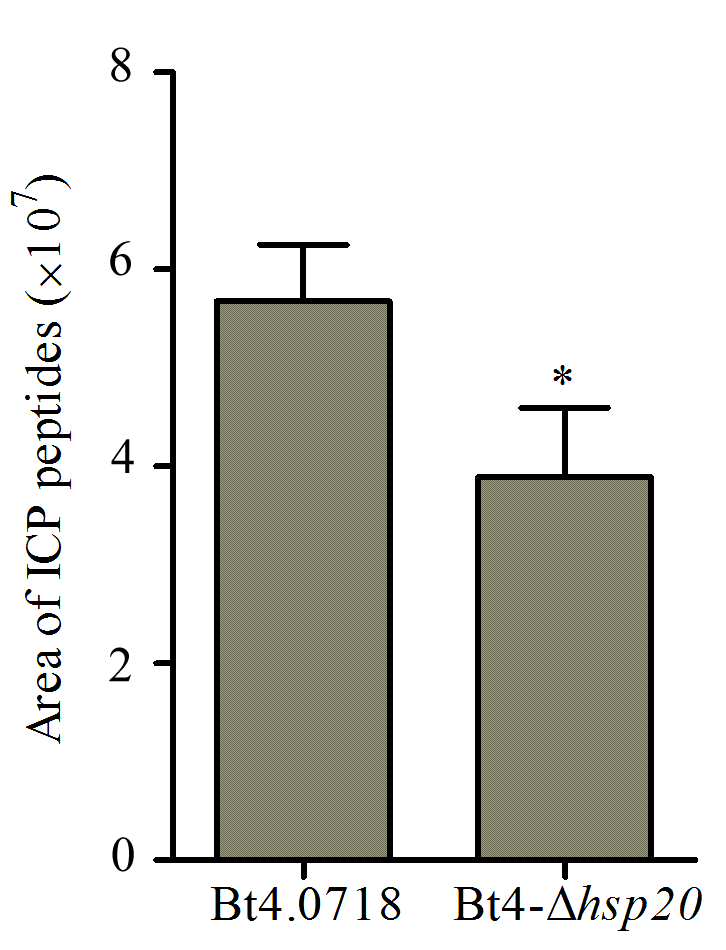

Supplement: FIGURE S4 — Peptides peak area of Cry1Ac and Cry1Aa in Bt4.0718 and hsp20 knockout strain (Std. Error = 0.210, ∗P < 0.05). [file Image_4.TIF]

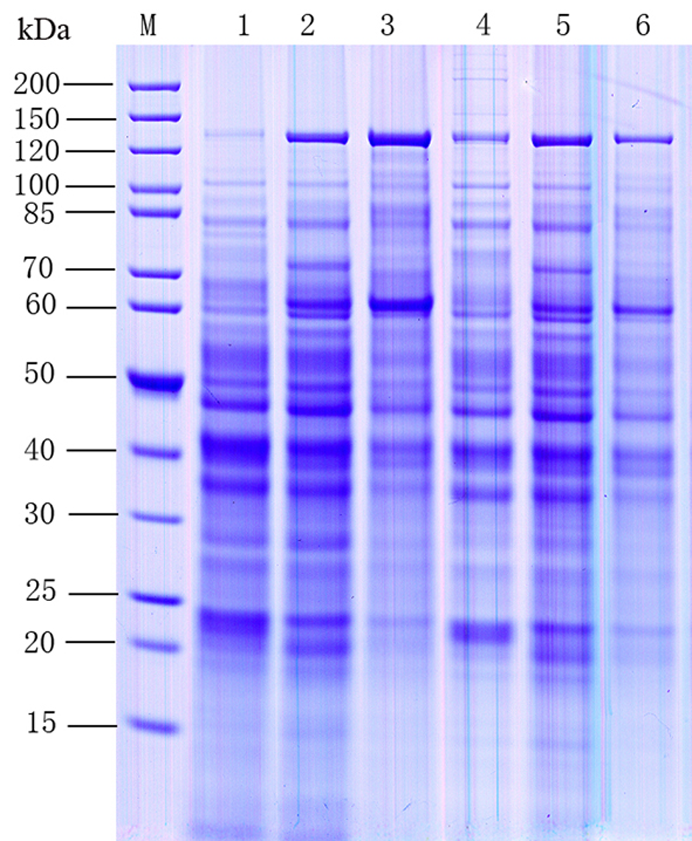

Supplement: FIGURE S5 — SDS-PAGE of the whole cell protein samples of Bt4.0718 and the hsp20 knockout strain at different time (lanes 1–3: 18, 24, and 28 h of Bt4.0718 strain; lanes 4–6: 18, 24, and 28 h of the hsp20 knockout strain). [file Image_5.TIF]
